# Antibacterial Superabsorbent Polymers from Tara Gum Grafted Poly(Acrylic acid) Embedded Silver Particles

**DOI:** 10.3390/polym10090945

**Published:** 2018-08-24

**Authors:** Mingfang Chi, Chang Liu, Jie Shen, Zhehai Dong, Zi Yang, Lijuan Wang

**Affiliations:** 1 Key Laboratory of Bio-Based Materials Science and Technology of Ministry of Education, Northeast Forestry University, Harbin 150040, China; 1057709686@nefu.edu.cn (M.C.); 18145145911@nefu.edu.cn (C.L.); sj@nefu.edu.cn (J.S.); 1007558214@nefu.edu.cn (Z.D.); 1024279298@nefu.edu.cn (Z.Y.); 2 Research Center of Wood Bionic Intelligent Science, Northeast Forestry University, Harbin 150040, China

**Keywords:** superabsorbent polymers, tara gum, silver, antibacterial properties

## Abstract

Tara gum/silver composite superabsorbent polymers were synthesized with tara gum grafted poly(acrylic acid), using K_2_S_2_O_8_ (KPS) as an initiator and *N*,*N*′-methylenebisacrylamide (MBA) as a cross-linker. The products were characterized by Fourier transform infrared spectroscopy (FTIR), X-ray diffraction (XRD), transmission electron microscope (TEM), scanning electron microscope (SEM) and X-ray photoelectron spectroscopy (XPS). The results showed that the silver ions were partially reduced to Ag^0^ and the amorphous nanoparticles containing Ag^0^ and Ag_2_O were around 10~50 nm in size The tara gum/silver composite superabsorbent polymers exhibited an interconnected porous structure with strong water absorption capacity. The swelling ratio of each product could reach 473 g/g in distilled water and 62 g/g in 0.9% NaCl solution. The antimicrobial activity of the samples against *Staphylococcus aureus* and *Escherichia coli* increased with the addition of AgNO_3_ from 0 to 125 mg. This work indicates that the developed tara gum/silver composite superabsorbent polymers can be potentially used for biomedical applications.

## 1. Introduction

Superabsorbent polymers are three-dimensional, cross-linked hydrophilic, linear or branched polymers [1]. They have the ability to absorb large quantities of water, saline and physiological solutions, compared with general absorbent materials [2]. Because of their high swelling ratio (SR) and biocompatibility, superabsorbent polymers have wide applications in biomedical engineering, agriculture and environmental protection [3].

There are many methods for the synthesis of superabsorbent polymers. The commercial water-absorbing resins are usually synthesized by free-radical solution polymerization via acrylic acid as the monomer, potassium persulfate (KPS) as the initiator and *N*′*N*-methylenebisacrylamide (MBA) as a crosslinking agent [4]. The product has good water absorption performance. However, the crosslinked poly(acrylic acid) cannot easily degrade under natural conditions due to its large molecular mass. According to the papers written by Edirisinghe et al. [5,6], the poly(methyl methacrylate) (PMMA) was dissolved in chloroform to produce the fibers by Pressurized Gyration technology. An effective method was used to process the soluble polymers into particles in large-scale production [7]. However, these two methods are not suitable for insoluble 3D polymers.

Researchers have improved the properties of superabsorbent polymers by grafting poly(acrylic acid) on biopolymers, to make them partially degrade under natural conditions. Safaa et al. [2] used gamma radiation to illuminate a mixture of tara gum (TG) and acrylic acid, in the presence of MBA as a crosslinking agent, to prepare hydrogels. TG is made from the endosperm of the seed *Caesalpinia spinosa*, which is a natural product used as a food additive that can degrade under natural conditions. It is a polysaccharide composed of galactomannan with high molecular mass, which can be induced by an initiator to produce free radicals. Therefore, TG is a good candidate for the synthesis of partially degradable superabsorbent polymers.

Nowadays, superabsorbent polymers are widely used in medicine and health care, and as a result products with antibacterial properties are in demand. To improve the antibacterial properties of superabsorbent polymers, metal particles can be added in the form of micro- or nanoparticles, such as gold [8], silver [9,10], iron [11], TiO_2_ [12] and carbon nanotubes [13]. Those composite superabsorbent polymers have been prepared through free-radical polymerization and grafting copolymerization [14] by using crosslinking [15] and reducing agents. Silver particles are favorite because of the antimicrobial efficacy against bacteria, fungi and viruses. Polymer composites containing silver particles have been widely used in wound dressings, scaffolds, water purification systems and medical devices [16]. The production methods of composite materials containing silver, such as gas condensation [17], electrochemical [18] and salt reduction [19], have been widely used. Wei et al. [20] synthesized nanosilver through ultraviolet irradiation. Mohan et al. [3] prepared hydrogels and then immersed them in silver ion solutions to synthesize silver-containing composite products. Through these methods, highly stable and uniformly distributed silver particles were produced in resins, however they require high energy and long periods of time. In previous works, different green reducing agents were employed to obtain Ag^0^ in hydrogels such as extract of blackberry [21] and cologynths seed [22]. As glucose is an edible and abundant monosaccharide widely used in many fields, it can easily reduce Ag^+^ ions to Ag^0^. To our best knowledge, it has not been reported that poly(acrylic acid) can be grafted onto tara gum molecules to form superabsorbent polymers embedded with Ag^0^ particles from Ag^+^ reduced by glucose.

In this paper, an easy, fast and energy-saving, one-pot method was employed to prepare TG/Ag composite superabsorbent polymers with excellent antibacterial abilities. To explore the effect of silver content on the antibacterial properties, we adjusted the silver nitrate solution amount and controlled the reduction rate to obtain four samples with 0, 5, 30 and 125 mg of AgNO_3_, labelled as Z0, Z1, Z2 and Z3 respectively. The characterizations were conducted with X-ray diffraction (XRD), transmission electron microscopy (TEM), scanning electron microscope (SEM), Fourier transform infrared spectroscopy (FTIR) and X-ray photoelectron spectroscopy (XPS). The effects of adding AgNO_3_ to the superabsorbent polymers on the swelling behavior in different aqueous media, and the antibacterial properties for Gram-negative *Escherichia coli* and Gram-positive *Staphylococcus aureus* bacteria, were investigated.

## 2. Materials and Methods

### 2.1. Materials

Tara gum, potassium persulfate (K_2_S_2_O_8_), sodium hydroxide (NaOH) were purchased from Tianjin Sitong Chemical Reagent Factory (Tianjin, China). Acrylic acid (AAc) was purchased from Tianjin Tianli Chemical Reagent Co. LTD (Tianjin, China). Cross-linker (*N*,*N*′-methylenebisacrylamide) was purchased from Tianjin Regent Chemical Co. LTD. Silver nitrate solution (AgNO_3_) was purchased from Shanghai Fine Chemical Material Research Institute. Glucose solution (C_6_H_12_O_6_) was purchased from Tianjin Yongda Chemical Reagent Co. LTD (Tianjin, China). Agar was purchased from Beijing Aoboxing Biotechnology Corporation (Beijing, China). *E. coli* (ATCC 23282) and *S. aureus* (ATCC 35696) bacterial strains were purchased from Qingdao Haibo Biotechnology Co. LTD (Qingdao, China). Distilled water and ethanol absolute (Liaoning Quanrui Reagent Co. LTD, Shenyang, China) were used as the solvent or for rinsing. All the chemicals were of analytical grade. The polymerization inhibitor in acrylic acid was removed by activated carbon while other chemicals could be used directly without any treatment.

### 2.2. Preparation of TG/Ag Composite Superabsorbent Polymers

A one-pot method was used to synthesize the silver-containing composite superabsorbent polymers. TG (1.0000 g) was dissolved in 20.00 mL of distilled water at 65.0 °C with continuous mechanical stirring. Six milliliters of K_2_S_2_O_8_ (1 g/50 mL) were then added to the solution as an initiator, and TG was triggered to produce free radicals. Subsequently, 28.40 mL of sodium hydroxide (8 g/100 mL), 6.00 mL of acrylic acid and 1.60 mL of cross-linker (1 g/100 mL) were mixed in an ice bath and added to the mixed solution. Silver nitrate solutions (1 g/100 mL) of different dosages (5, 30 and 125 mg) and a proportionate glucose solution (2 g/100 mL) were added into the system at 65.0 °C for 1 h to reduce Ag^+^ into silver particles. The product was washed with ethanol absolute until there was no water remaining and then dried by ordinary, hot air drying for 12 h at 60.0 °C. Finally, the dry samples were crushed into powder and sifted for subsequent experiments and tests.

### 2.3. Characterization Methods

The functional groups were analyzed with a Fourier transform infrared spectrometer (NICOLET AVATAR360, Thermo Fisher Scientific Co., Ltd., Waltham, MA, USA) in a wavenumber range of 4000–400 cm^−1^ with a resolution of 4 cm^−1^. XRD patterns were collected with an X-ray diffractometer (BRUKERD8-ADVANCE, Berlin, Germany; Cu Kα radiation (*k* = 1.5418 Å), voltage: 40 kV, current: 40 mA, scanning range: 5–90°). XRD was mainly used to identify the silver particles in the superabsorbent polymers. XPS was performed by using a K-Alpha 89 (Thermo Electron, London, UK) to identify the silver particles in the superabsorbent polymers. The TG/Ag composite superabsorbent particles were dispersed in ethanol and cast on an ultrathin, carbon supporting film, then dried at room temperature. TEM measurements were performed on a TEM, JEM2100 (JEOL LTD, Tokyo, Japan). After coating with gold, the morphology of freeze-dried samples was observed with a Hitachi S-4800 field-emission scanning electron microscope (FEI Nova NanoSEM 450, Los Angeles, CA, USA) (SEM) equipped with energy dispersive spectrum (EDS).

### 2.4. Antibacterial Test

The antibacterial activity of the TG/Ag composite superabsorbent polymers against both *E. coli* (Gram-negative) and *S. aureus* (Gram-positive) was tested according to an agar diffusion test. For the agar diffusion method, samples were exposed to bacteria in solid media (nutrient agar), and the inhibition zone around each sample was measured and to record the antibacterial effect of the Ag particles. The bacterial suspension was inoculated to the agar plates for a bacteriostatic test. The sample powder was pressed into sheets with a diameter of 1 cm and placed on the agar plate, then incubated at 37 °C for 24 h. The size of the inhibition zone was measured to detect the antibacterial properties.

### 2.5. Swelling Studies

A quantity of 0.2 g of powdered TG/Ag composite superabsorbent polymers was immersed in 200 mL of distilled water and 100 mL of 0.9% NaCl, respectively. After 2 h, it was taken from the solution and hung for 0.5 h before its weight was measured. The *SR* of the superabsorbent polymers was recorded during swelling as indicated in Equation (1).

*SR* = (*W*_s_ − *W*_d_)/*W*_d_ (1)

where *W*_d_ is the initial weight of the sample and *W*_s_ is the weight of the sample after swelling. All experiments were carried out in triplicate.

## 3. Results and Discussion

### 3.1. The Networks of TG/Ag Composite Superabsorbent Polymers

**Figure 1:**
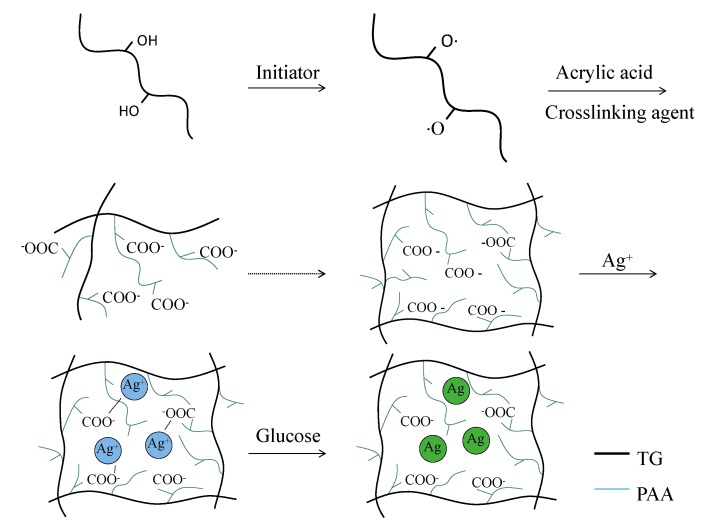
The schematic procedure of Ag particle formation in TG/Ag superabsorbent network.

The schematic procedure of Ag particle formation in TG/Ag superabsorbent network is shown in Figure 1. Monomer polymerization was caused by the initiator and long chains were interwoven into reticulation by the cross-linker. As the reducing agent, glucose was responsible for the reduction of Ag^+^ to embed it into the network. The slight excess of the reducing agent was necessary to favor the formation of monodispersed metal through the fast nucleation process [23]. Finally, the ratio of glucose to silver nitrate was 2:1.

### 3.2. FTIR Spectroscopy

**Figure 2:**
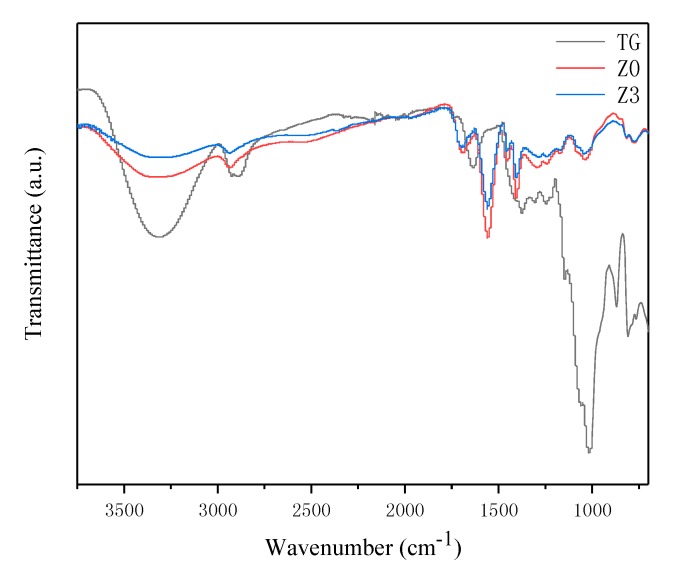
FTIR spectra of the TG, Z0 and Z3.

Figure 2 shows the FTIR spectra of TG, Z0 and Z3. The spectrum of TG presents a band at ~3300 cm^−1^ which is attributable to –OH stretching vibration. The band at around 2800–3000 cm^−1^ corresponds to C–H stretching vibration. A series of peaks at 1040, 1065, 1120, and 1168 cm^−1^ correspond to –C–O–C– bonds in the anhydroglucose unit in TG. After grafting poly(acrylic acid), the spectrum (Z0) was similar to TG, except that the characteristic bands of –OH group and anhydroglucose unit decreased. A new band occurred at ~1700 cm^−1^ corresponding to the stretching vibration in the carboxylate and carboxylic acid groups, respectively. The results show that poly(acrylic acid) chains were successfully grafted on TG molecules. After embedding Ag particles, the spectrum (Z3) did not obviously change.

### 3.3. Size and Morphology of Ag Particles

#### 3.3.1. XRD Analysis

**Figure 3:**
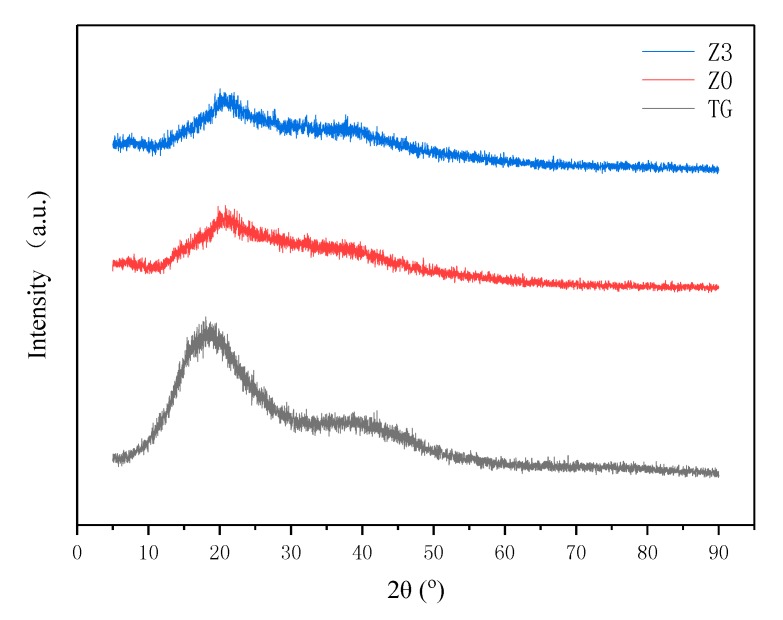
X-ray diffraction pattern of TG, Z0 and Z3.

Figure 3 shows the XRD patterns of TG, Z0 and Z3. The TG has a broad peak at ~19° indicating amorphous and crystalline regions existed because a large amount of –OH groups interacted via intermolecular hydrogen bonds. After grafting, the peak strength decreased, which indicated that intermolecular hydrogen bonds were damaged. After embedding Ag particles, the XRD pattern is similar as that of Z0. In particular, no obvious peak of Ag occurred, which indicated that no Ag particle or crystalline Ag particles formed in the network of the superabsorbent polymers.

#### 3.3.2. TEM Analysis

**Figure 4:**
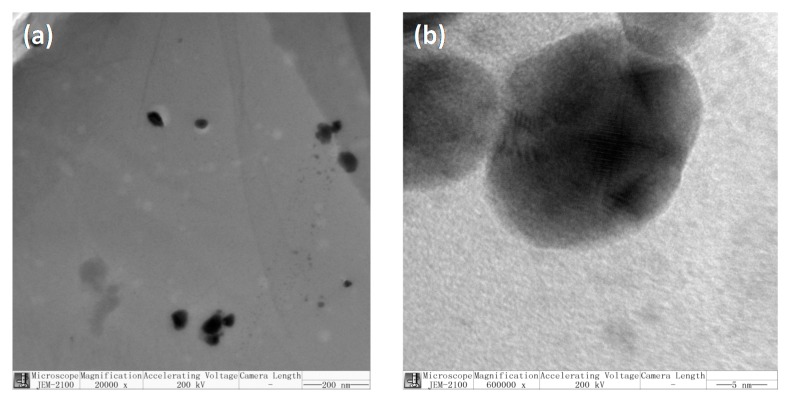
TEM micrographs of Z3, (**a**) 20,000× and (**b**) 600,000×.

TEM photographs of the superabsorbent polymers (Z3) are shown in Figure 4. Ag particles are present in irregular nanosphere form with the size of around 10~50 nm. The nanospheres varied in size and obvious aggregation occurred among some spheres. The results indicated that the Ag nanoparticles were successfully embedded in the superabsorbent polymers. 

#### 3.3.3. XPS Spectroscopy

**Figure 5:**
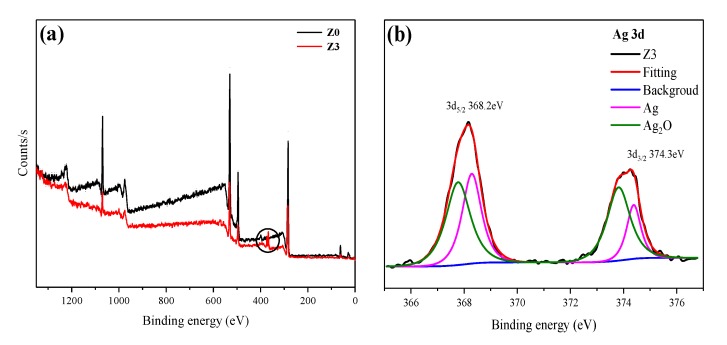
(**a**) XPS wide region scan spectra of the Z0 and Z3. (**b**) High resolution XPS Ag 3D spectrum of Z3.

XPS spectra of Z0 and Z3 were shown in Figure 5. The peaks of O, C and N elements were found on the wide scan XPS spectrum of Z0. A new peak at ~370 eV was found on the spectrum of Z3 compared with Z0. It can be seen from Figure 5b that the high-resolution Ag 3d_3/2_ and Ag 3d_5/2_ spectrum of Z3 can be approximately decomposed into two peaks at 374.4 and 373.8 eV, 368.3 and 367.8 eV. This could be ascribed to Ag and Ag_2_O, respectively [24]. Therefore, Ag and Ag_2_O formed in the superabsorbent. The results further proved that the reduction rate of silver is less than 100%. Ag_2_O is also an antibacterial agent [25], which could increase the antibacterial performance of the product.

#### 3.3.4. SEM Analysis

**Figure 6:**
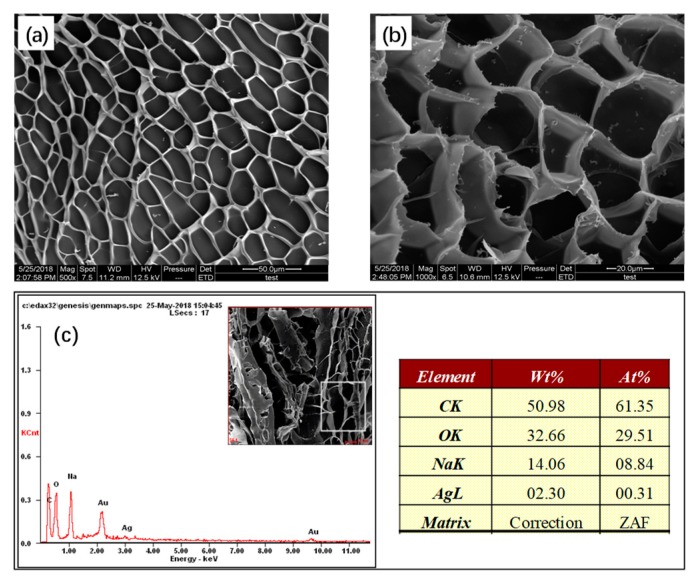
SEM micrographs (**a**) 500×, (**b**) 1000× and EDS (**c**) of Z3.

Figure 6a,b show that superabsorbent polymers has a developed 3D network structure which provides space for water absorption and water conservation. The size of pores are about 20~30 nm, which is the size of ice crystals removed by freeze-drying. Figure 6c shows that the product contains a small amount of Ag-derived component and a large amount of Na^+^. This provides the osmotic pressure for enhancing the water absorption, giving the resin salt-resistance and an Ag-derived component providing an antibacterial basis. The existence of two ions both provides a basis for biomedical applications.

### 3.4. Antibacterial Properties of TG/Ag Composite Superabsorbent Polymers

**Figure 7:**
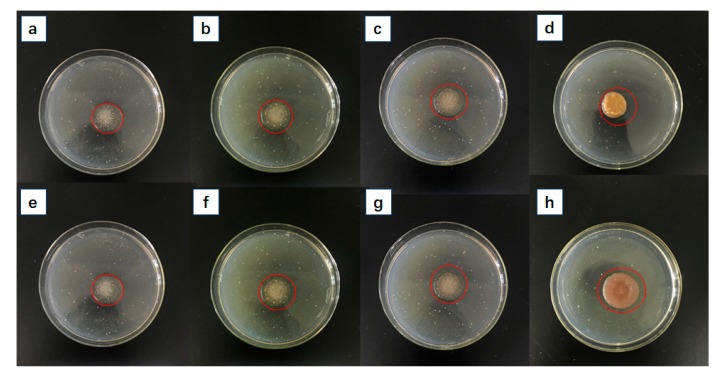
Inhibition zone photographs of Z0, Z1, Z2 and Z3 against bacteria *S. aureus* (**a**–**d**) and *E. coli* (**e**–**h**)*.*

The in vitro antibacterial properties of the TG/Ag composite superabsorbent polymers were compared against Gram-negative *E. coli* and Gram-positive *S. aureus* bacteria with a disk diffusion test. The inhibition zone under and around the tested samples for bacterial growth was detected visually and is listed in Table 1 and shown in Figure 7. The results show that Ag-free superabsorbent gave 2.4 cm of bacteriostatic ring for *S. aureus* and *E. coli*. The antibacterial efficiency of the composite superabsorbent polymers increased with the addition of AgNO_3_ increasing regardless of the kind of bacterial used. Z3 which had the highest antibacterial performance.

### 3.5. Swelling Behavior in Distilled Water and 0.9% NaCl Solution

The swelling ratio of the superabsorbent polymers are shown in Table 2. It reached 473 g/g in distilled water and 62 g/g in 0.9% NaCl solution. With an increase of AgNO_3_ from 5 to 125 mg, the swelling ratio reduced from 332 to 273 g/g because more silver particles in the network blocked the swollen behavior. The results suggest that the superabsorbent polymers containing silver particles had good water absorption capacity and antibacterial performance.

## 4. Conclusions

In this study, a fast and simple method was used to prepare antibacterial superabsorbent polymers from tara gum grafted poly(acrylic acid) embedded silver particles, by using glucose as reducing agent. Different dosages of silver nitrate and glucose solutions were added to produce silver in the net structure to achieve different antibacterial effects. FTIR analysis showed that poly(acrylic acid) chains were successfully grafted on TG molecules. XPS and XRD results indicated that amorphous Ag and Ag_2_O formed in the product and the reduction rate of silver was less than 100%. The TEM results indicate that the nanoparticles consisting of Ag and Ag_2_O were successfully embedded in the superabsorbent polymer, and particles are present in irregular nanosphere form with the size of around 10~50 nm. The SEM results showed that the obtained polymers had a 3D network structure and pores of about 10~20 nm, which favored water absorption. The products had good antibacterial properties and the bacteriostatic effect improved with increasing silver content. The product could absorb 273 g/g of distilled water and 37 g/g water in 0.9% NaCl solution, when 125 mg of AgNO_3_ was added. Based on these findings, the TG/Ag composite superabsorbent polymers could hopefully be used in different medical fields, such as drug delivery, wound healing and tissue engineering.

## Figures and Tables

**Table 1:** The diameter of the bacteriostasis circle of the samples.

Sample	AgNO_3_ (mg)	Bacteriostatic Ring of *S. aureus* (cm)	Bacteriostatic Ring of *E. coli* (cm)
Z0	0	2.4	2.4
Z1	5	2.6	2.5
Z2	30	2.8	2.7
Z3	125	3.5	3.2

**Table 2:** The effects of AgNO_3_ amounts on the swelling ratio of the samples.

Sample	AgNO_3_ (mg)	Absorption of Water (g/g)	Absorption of Water in 0.9% NaCl Solution (g/g)
Z0	0	473	62
Z1	5	332	51
Z2	30	324	49
Z3	125	273	37
